# Oxidized LDL in Inflammation: From Bench to Bedside

**DOI:** 10.1155/2013/762759

**Published:** 2013-11-24

**Authors:** Ishak Ozel Tekin, Asım Orem, Ronit Shiri-Sverdlov

**Affiliations:** ^1^Department of Immunology, Bulent Ecevit University, School of Medicine, 67600 Zonguldak, Turkey; ^2^Department of Biochemistry and Clinical Biochemistry, Karadeniz Technical University, School of Medicine, 61080 Trabzon, Turkey; ^3^Department of Molecular Genetics, Maastricht University (MUMC), Universiteitssingel 50, 6229 ER, Maastricht, The Netherlands

Oxidation of LDL is considered a marker of inflammation. Indeed, numerous studies have documented that oxidized lipids as well as products derived from their decomposition have deleterious biological properties. As such a better understanding of the underlying pathological mechanisms is necessary for the development of novel therapeutic agents and diagnostic tools. Multiple experimental data carried out in cellular lines and animal models of atherosclerosis support the proatherogenic role of oxidized LDLs through several mechanisms which include chemotactic and proliferating actions on macrophages, stimulation of smooth muscle cells, and eliciting apoptosis. While the role of oxLDL in relation to atherosclerosis has been investigated thoroughly, its role in other inflammatory diseases is recently emerging as well. 

In this special issue, we report how findings regarding oxLDL in inflammation at the benchside can be translated to the bedside and how these observations may help clinical practice. The papers have been contributed by a number of experts in the field and include both review articles that provide an overview of the work conducted to date and original articles reporting recent discoveries and innovations. In order to highlight the translational relevance, several papers are focused on novel basic mechanisms as well as clinical evidence related to atherosclerosis. In addition, papers describing the novel link between oxLDL to COPD, endometriosis, and preeclampsia are included. We hope that this series of papers will be beneficial for clinicians and researchers in their diagnostic and therapeutic approaches towards inflammatory diseases. Each of the papers in this series is briefly highlighted as follows.

G. Maiolino et al. in the review “*The role of oxidized low-density lipoproteins in atherosclerosis: the myths and the facts*” examine the role played by oxidized LDLs in atherogenesis taking into account data derived by studies based on molecular and clinical approaches. The authors provide available data from animal models and patients cohorts that validate the oxidative modification hypothesis of atherosclerosis. Several experimental papers in this special issue support this view. The paper by F. Mascarenhas-Melo et al. “*Implication of low HDL-c levels in patients with average LDL-c levels: a focus on oxidized LDL, large HDL subpopulation, and adiponectin*” demonstrates that in a patient population with cardiovascular risk factors low HDL-c levels are associated with a poor cardiometabolic profile, despite the average levels of LDL-c. This data suggests that therapeutic interventions directed to inhibition of Ox-LDL levels and raising HDL-c levels and functionality are advisory preventive measures in this type of cardiovascular risk populations.

Furthermore, J.-F. Zhao et al. in “*Activation of TRPV1 prevents OxLDL-induced lipid accumulation and TNF-*α*-induced inflammation in macrophages: role of liver X receptor *α**” demonstrate that suppression of oxLDL and activation of LXR*α* by TRPV1 is protective in macrophages. These findings may help in developing novel pharmacological targets for treating atherosclerosis-related cardiovascular diseases.

Finally, J. Li et al. in “*Minimally modified LDL upregulates endothelin type A receptors in rat coronary arterial smooth muscle cells*” investigated the effects of mmLDL on the expression of endothelin type A (ETA) receptors in coronary arteries. The authors demonstrated that mmLDL contributes to the development of ischemic cardiovascular diseases by inducing an upregulation of ETA receptors in the coronary artery. The molecular mechanisms involve the activation of PKC and ERK1/2 MAPK pathways and the downstream NF-*κ*B signalling pathways. 

The lectin-like oxidized low-density lipoprotein receptor-1 (LOX-1) is the main OxLDL receptor of endothelial cells, and it is expressed also in macrophages and smooth muscle cells. A. Pirillo et al. in “*LOX-1, OxLDL, and atherosclerosis*” describe the evidence from mouse and humans data, suggesting that LOX-1 might be an attractive therapeutic target for the management and the prevention of atherosclerosis. Inhibition of the LOX-1 receptor is currently being investigated and represents an emerging approach for controlling OxLDL-LOX-1-mediated proatherogenic effects. The important role of oxidized LDL and LOX-1 in inflammation is further discussed in the review “*Oxidized LDL and LOX-1 in experimental sepsis*” by N. Al-Banna and C. Lehmann. This review highlights the evidence relating oxLDL and LOX-1 to proinflammatory disease mechanisms. Situations in which oxLDL is involved in disease resolution due to exposure time, dose, or degree of oxidization are described. The emerging role of LOX-1 in the migration capacity of MSCs is demonstrated by F. Zhang et al. in “*Ox-LDL promotes migration and adhesion of bone marrow-derived mesenchymal stem cells via regulation of MCP-1 expression*.” The authors investigated the effects of ox-LDL on bone marrow-derived mesenchymal stem cells (bmMSCs) migration and adhesion as well as the related mechanisms. They concluded that ox-LDL-induced bmMSC migration and adhesion are dependent on LOX-1 activation and MCP-1 expression. This study is important since the migration capacity of MSCs is one of the determinants of the efficiency of MSC-based transplant therapy.

The dual biological effect of electronegative low-density lipoprotein (LDL (−)) which is the minor modified fraction of LDL found in blood is presented in the review “*Electronegative LDL: a circulating modified LDL with a role in inflammation*” by M. Estruch et al. This review compares LDL (−) to oxLDL, updates findings on the inflammatory and anti-inflammatory effects of LDL (−) on cells, and discusses its putative physiological role.

M. Gursel et al. in “*Plasmacytoid dendritic cell response to CpG ODN correlates with CXCL16 expression and is inhibited by ox-LDL*” report that ox-LDL presence significantly inhibited D-ODN mediated IFN*α* production by plasmacytoid dendritic cells. Moreover the authors demonstrated that CXCL16/CXCR6 interaction can modify the response of pDCs to environmental danger signals.

F.J. Rios et al. in “*Oxidized LDL induces alternative macrophage phenotype through activation of CD36 and PAFR*” examined the effect of oxLDL on macrophage phenotype. The authors concluded that oxLDL induced macrophage differentiation and activation towards the alternatively activated (M2) phenotype. Furthermore, they showed that this profile of macrophage activation is dependent on the engagement of both CD36 and PAFR. 

Among the different models of LDL oxidation that have been studied, the one using myeloperoxidase (MPO) is thought to be more physiopathologically relevant. K. Z. Boudjeltia et al. in “*Myeloperoxidase-dependent LDL modifications in bloodstream are mainly predicted by angiotensin II, adiponectin, and myeloperoxidase activity: a cross-sectional study in men*” suggest that the combination of blood MPO activity, angiotensin II, and adiponectin explains, at least partially, serum Mox-LDL levels. This is significant since serum Mox-LDL levels are involved in the pathogenesis of atherosclerosis. Another example for the important role of LDL oxidation by myeloperoxidase (Mox-LDL) in the context of atherogenesis is presented by C. Delporte et al. in the review “*Low-density lipoprotein modified by myeloperoxidase in inflammatory pathways and clinical studies.*” The review focuses on activation of endothelial cells and monocytes/macrophages, induction of proinflammatory molecules, and inhibiting of fibrinolysis as the main mechanisms by which Mox-LDL increases the risk of thrombus formation. Moreover, the role of Mox-LDL in the physiopathology of several diseases linked to atherosclerosis is also discussed. In addition to atherosclerosis, the involvement of oxLDL in other inflammatory diseases has recently emerged. G. Polak et al. in “*Low-density lipoproteins oxidation and endometriosis*” found that concentrations of oxLDL in peritoneal fluid of endometriotic women were significantly higher compared to women with serous but not dermoid ovarian cysts. Their results indicate that disrupted oxidative status in the peritoneal cavity of women with endometriosis may play a role in the pathogenesis of advanced stages of the disease. The possible relationship between COPD and ox-LDL has been investigated recently by Y. Shen et al. in “*Increased serum ox-LDL levels correlated with lung function, inflammation, and oxidative stress in COPD.*” The authors describe that serum levels of ox-LDL are increased in COPD patients and are correlated with reduced lung function, inflammation, and oxidative stress in COPD. Ş. Açikgöz et al. in “*Levels of oxidized LDL, estrogens, and progesterone in placenta tissues and serum paraoxonase activity in preeclampsia*” demonstrate that the events leading to fetoplacental insufficiency lead to a reduction in the levels of estriol limit deposition of OxLDL in placental tissues. These observations point towards the importance role of serum PON1 activity in the inhibition of OxLDL in preeclampsia. 

Finally, “*Paraoxonase-1 inhibits oxidized low-density lipoprotein-induced metabolic alterations and apoptosis in endothelial cells: a nondirected metabolomic study*” summarized by A. García-Heredia et al. showed alterations in carbohydrate and phospholipid metabolism and increased apoptosis in cells incubated with oxidized LDL. These results extend current knowledge on the protective role of HDL and PON1 against oxidation and apoptosis in endothelial cells.

We sincerely hope that the present special issue may provide useful information to understand the mechanisms, the clinical effects, and the novel treatments of oxLDL-induced inflammation. We hope that the reader will find some novel input for future researches.

